# Inhibitory Action of Quercetin on Eosinophil Activation *In Vitro*

**DOI:** 10.1155/2013/127105

**Published:** 2013-06-06

**Authors:** Misako Sakai-Kashiwabara, Kazuhito Asano

**Affiliations:** ^1^Graduate School of Nursing and Rehabilitation Sciences, Showa University Graduate School, Yokohama, Kanagawa 226-8555, Japan; ^2^Bio-Thera Clinic, Shinjuku, Tokyo 162-0022, Japan; ^3^Division of Physiology, School of Nursing and Rehabilitation Sciences, Showa University, Yokohama, Kanagawa 226-8555, Japan

## Abstract

The influence of quercetin on eosinophil functions was examined *in vitro* and *in vivo*. The first set of experiments was undertaken to examine whether quercetin could suppress eosinophilia and IgE hyperproduction induced by *Mesocestoides corti* infection in BALB/c mice. The number of peripheral blood eosinophils and IgE levels were examined 21 days after infection. Oral administration of quercetin for 21 days could not suppress both peripheral blood eosinophilia and IgE hyperproduction, even when 20.0 mg/kg quercetin was used for treatment. The second part of the experiment was designed to examine the influence of quercetin on eosinophil activation induced by SCF stimulation *in vitro*. Eosinophils were obtained from *M. corti*-infected mice and stimulated with SCF in the presence of various concentrations of quercetin for 24 h. The addition of quercetin into cell cultures could suppress eosinophil activation induced by SCF stimulation as assessed by measuring the contents of RANTES, MIP-1**β**, ECP, and MBP in culture supernatants. The minimum concentration of quercetin which caused significant suppression of factor secretion was 5.0 **μ**M. These results may suggest that quercetin will be a good candidate for the supplement on the management of eosinophil-mediated diseases, such as allergic rhinitis and asthma.

## 1. Introduction 

The inflammatory responses in airway diseases such as allergic rhinitis and asthma involve a complex network of several inflammatory cells and mediators. These include antigen-presenting cells, Th2 type helper T cells, mast cells, eosinophils, and fibroblasts [1, 2]. Although mast cells and Th2 type helper T cells largely contribute to the induction and the early phase of response of the allergic reaction, eosinophils are believed to be the main type of cells recruited during the late phase and play an essential role as final effector cells in the development of persistent inflammation and tissue damages through the secretion of cationic proteins, lipid mediators, cytokines, and chemokines [2, 3]. This was confirmed by histological examination, which showed extensive degranulation of eosinophils in airway tissues during active diseases [4]. The presence of much higher levels of granule proteins in the damaged tissues is also observed [5]. These reports may suggest that the manipulation of eosinophil functions, such as activation and degranulation, will be a good therapeutic target in the treatment and prevention of allergic diseases.

Quercetin belongs to a group of plant compounds called flavonoids, which give many fruits, flowers, and vegetables their color [6]. For many years, quercetin has been studied for the possible health benefits, and it has been revealed that quercetin plays the role of scavenger for free radicals, which damage cell membrane, tamper with DNA, and even cause cell death [7]. It is also reported that quercetin can inhibit the release of histamine and other mediators responsible for the development of allergic reaction from mast cells [8, 9]. Furthermore, oral administration of quercetin is reported to attenuate the clinical symptoms (e.g., bronchial hyperreactivity among others) observed in a murine [10] and guinea pig asthma model [11]. However, the influence of quercetin on eosinophil functions is poorly understood.

Infection with parasites, except protozoa, is well known to induce IgE hyperproduction and peripheral blood eosinophilia in mammalian hosts [12, 13]. Because these immune responses are quite similar to those in allergic diseases [12, 13], the parasite/host system may be considered to provide a suitable model to examine the therapeutic mechanisms of antiallergic agents. *Mesocestoides corti* is a common parasite in dogs and humans in North and Central America. The larval worm of this parasite is called tetrathyridium larva and has been observed in the peritoneal cavity of wild rodents. Tetrathyridium larva infection in rodents is accepted to produce peripheral blood eosinophilia and IgE hyperproduction [12].

In the present study, therefore, we have used the *M. corti*/mouse system and examined the influence of quercetin on both peripheral blood eosinophilia and IgE hyperproduction *in vivo*. We also examined the influence of quercetin on the activation of eosinophils obtained from mice infected with *M. corti* by using an* in vitro *cell culture technique.

## 2. Materials and Methods

### 2.1. Mice

Specific pathogen-free male BALB/c mice, 5 weeks of age, were purchased from Charles River Japan Inc. (Atsugi, Japan). They were maintained in our animal facilities under a controlled environment (25 ± 3°C, 55 ± 5% humidity, and a 12 h light/dark cycle). All animal experimental procedures were approved by the Animal Care and Use Committee of Showa University and were carried out in accordance with the guidelines of the Physiological Society of Japan [12, 14].

### 2.2. Agent and Treatment

Quercetin was purchased from Sigma Chemicals Co. Ltd. (St Louise, MO, USA). For *in vivo* use, quercetin was well mixed with 5% gum tragacanth solution. The mice (5 mice/group) were given various doses (5.0, 7.5, 10.0, 15.0, 17.0, and 20.0 mg/kg) of quercetin once a day for 3 weeks via a stomach tube in a volume not exceeding 0.25 mL, starting on the day of infection. The control mice were administered orally with 5% of gum tragacanth solution alone. For *in vitro* used, quercetin was dissolved in dimethyl sulfoxide at a concentration of 1.0 M, then diluted at RPMI-1640 medium (sigma chemicals co. ltd.), and supplemented with 10% fetal calf serum (Nihon Bio-Supply Center, Tokyo, Japan; RPMI-FCS) at appropriate concentrations for experiments.

### 2.3. Parasitological Technique

*M. corti* kindly donated by Dr. A. Niwa (School of Medicine, Kinki University, Osaka, Japan) was maintained in mice by intraperitoneal injection of 500 tetrathyridia according to the method described previously [12].

### 2.4. Assay for IgE

Blood was obtained from retro-orbital plexus in a volume of 100 *μ*L. After clotting, the serum was obtained, and the total IgE levels were assayed by mouse IgE ELISA test kits (Yamasa Co. Ltd., Chiba, Japan). The ELISA was done in duplicate according to the manufacturer's recommended instructions. The minimum detectable level of these kits is 10.0 ng/mL.

### 2.5. Counting for Peripheral Blood Eosinophils

The number of eosinophils in peripheral blood was examined according to the method described previously [14]. Briefly, 5 *μ*L of the blood taken from retro-orbital plexus was mixed with 20 *μ*L of Hinkelman's solution (muto pure chemicals co. ltd., Tokyo, Japan). Eosinophils were counted using haemocytometers in triplicate. 

### 2.6. Culture of Eosinophils

Mice were killed by ether anesthesia 21 days after intraperitoneal injection with 500 tetrathyridia. Peritoneal cells were obtained by washing the mouse peritoneal cavity with 10 mL of sterile phosphate buffered saline. The cells were washed 3 times with RPMI-FCS and incubated in plastic tissue culture plates to remove plastic adherent cells in a humidified atmosphere with 5% CO_2_ at 37°C. After two hours, nonadherent cells (eosinophils) were collected and suspended in RPMI-FCS at a concentration of 5 × 10^5^ cells/mL. Eosinophils (1.0 mL) were then treated with various doses of quercetin for one hour and stimulated with 200.0 ng/mL stem cell factor (R & D Corp., Minneapolis, MN, USA) for 24 h in a final volume of 2.0 mL [14]. The culture supernatants were collected after pelleting cells by centrifugation at 3000 rpm for 15 min at 25°C and stored at −40°C until used. The purity of eosinophils was >95% as judged by Giemsa stain. 

### 2.7. Assay for Eosinophil-Derived Chemokines

Levels of eosinophil-derived chemokines, regulated on activation, normal T-cell expressed and secreted (RANTES) and macrophage inflammatory protein-1beta (MIP-1*β*), in culture supernatants were examined by commercially available ELISA test kits (R & D Corp.). The ELISA was done in duplicate according to the manufacturer's recommendations. The minimum detectable levels of these ELISA kits were 2.0 pg/mL and 1.5 pg/mL, respectively.

### 2.8. Assay Degranulation

Levels of eosinophil granule proteins, eosinophil cationic protein (ECP), and major basic protein (MBP) in culture supernatants were examined by commercially available ELISA test kits, which were purchased from Cusabio Biotech Co., Ltd. (Wuhan, China) and Uscn Life Science Inc. (Wuhan, China), respectively. The minimum detectable levels were 0.156 ng/mL for ECP and 0.225 ng/mL for MBP. The ELISA was done in duplicate according to the manufacturer's recommendations.

### 2.9. Statistical Analysis

Data were analyzed with analysis of variance (ANOVA) followed by Bonferroni test. *P* < 0.05 was considered statistically significant.

## 3. Results

### 3.1. Effect of Quercetin on Eosinophilia and IgE Hyperproduction Induced by *M. corti* Infection

**Figure 1:**
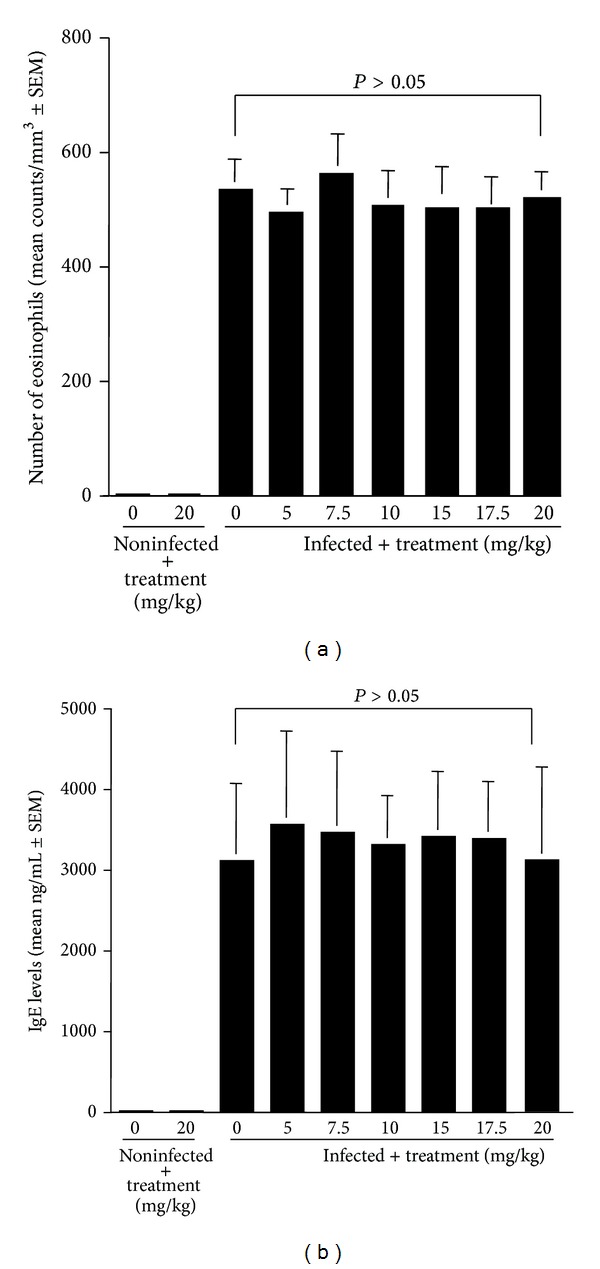
Influence of quercetin on peripheral blood eosinophilia (a) and IgE hyper-production (b) induced by *Mesocestoides corti* infection in mice. BALB/c mice were injected intraperitoneally with 500 *M. corti* larvae. These mice were then treated with various doses of quercetin once a day for 21 days, starting on the day of infection. After 21 days, the number of peripheral blood eosinophils and IgE levels were examined. Values are means ± standard errors of the mean (SEM) for five mice/group.

The first set of experiments was carried out to examine the influence of quercetin on eosinophilia and IgE hyperproduction. BALB/c mice were infected intraperitoneally with 500 *M. corti* larvae on day 0. These mice were then treated with various doses of quercetin once a day for 21 days, starting on the day of infection. The number of peripheral blood eosinophils and IgE levels were examined 21 days after infection. As shown in Figure 1(a), quercetin could not suppress the increase in the number of eosinophils induced by *M. corti* infection, even when the mice were treated with quercetin at more than 15 mg/kg/day. We then examined the influence of quercetin on IgE production caused by *M. corti* infection. The data in Figure 1(b) clearly showed the negative suppressive effect of quercetin on IgE production: the IgE levels in serum from mice treated with quercetin at 20 mg/kg were nearly identical (not significant) to that from nontreated, *M. corti*-infected mice. 

### 3.2. Influence of Quercetin on Eosinophil Activation *In Vitro*

**Figure 2:**
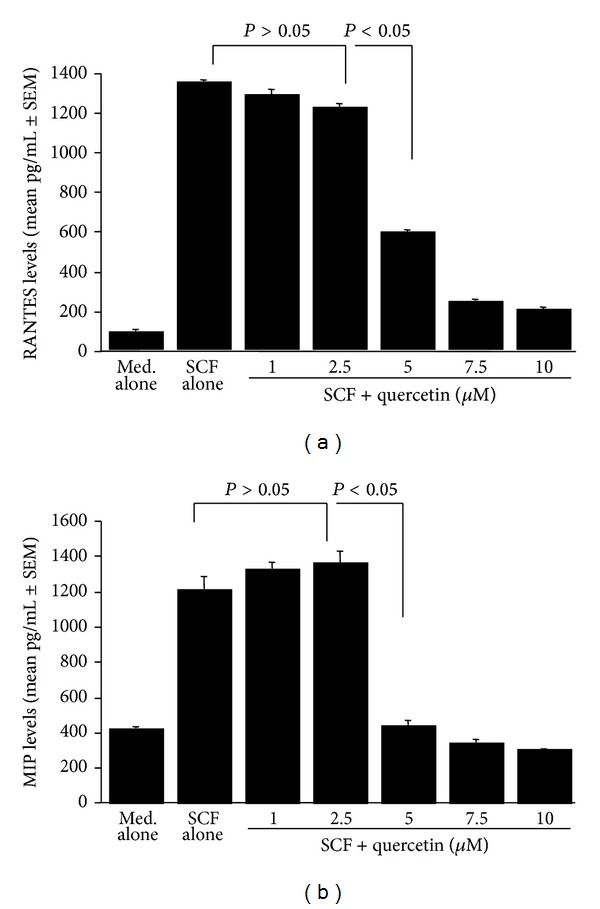
Influence of quercetin on eosinophil activation induced by stem cell factor (SCF) stimulation *in vitro*. Eosinophils (5 × 10^5^ cells/mL) obtained from mice infected with *Mesocestoides corti* (500 larvae/mouse) were stimulated in triplicate with 200 ng/mL SCF in the presence of various concentrations of quercetin for 24 h. Factor levels in culture supernatants were examined by ELISA. The data are expressed as the mean ± standard errors of the means (SEM). Med. alone: medium alone; (a) RANTES; (b) MIP-1*β* (MIP).

The second set of experiments was designed to examine whether quercetin could suppress eosinophil activation in response to immunological stimuli. To do this, eosinophils were pretreated with various doses of quercetin for one hour and then stimulated with 200.0 ng/mL SCF. After 24 h, culture supernatants were collected, and RANTES and MIP-1*β* levels were examined by ELISA. Quercetin could inhibit the ability of eosinophils to produce RANTES (Figure 2(a)) and MIP-1*β* (Figure 2(b)), which was enhanced by SCF stimulation. The minimum concentration of quercetin, which causes significant suppression of factor productions, was 5.0 *μ*M (Figures 2(a) and 2(b)). 

### 3.3. Influence of Quercetin on Eosinophil Degranulation *In Vitro*

**Figure 3:**
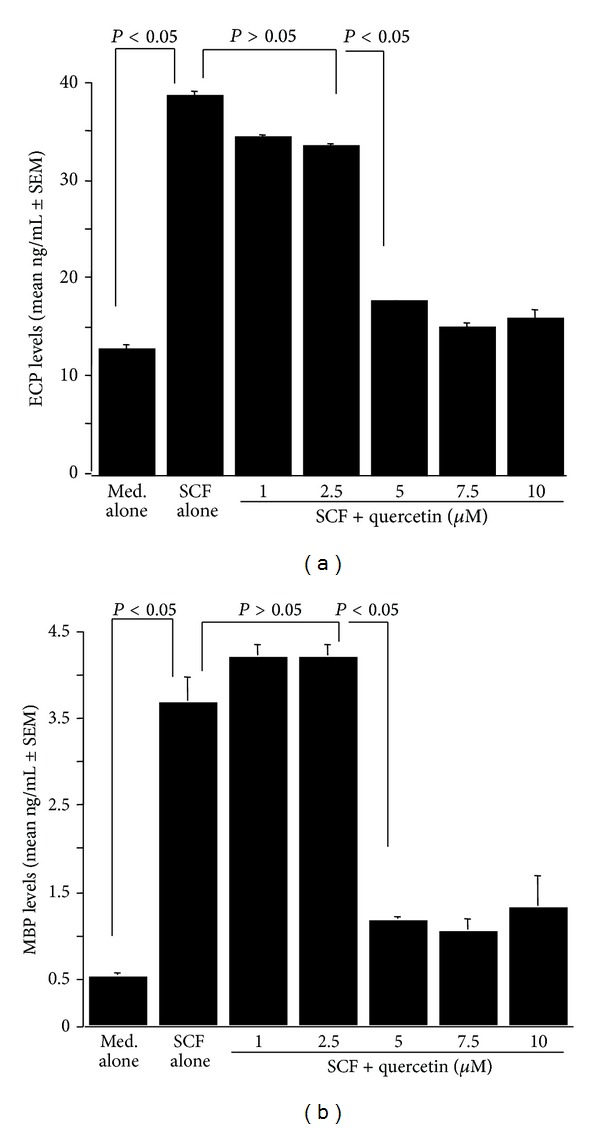
Influence of quercetin on eosinophil degranulation induced by stem cell factor (SFC) stimulation *in vitro*. Eosinophils (5 × 10^5^ cells/mL) obtained from mice infected with *Mesocestoides corti* (500 larvae/mouse) were stimulated in triplicate with 200 ng/mL SCF in the presence of various concentrations of quercetin for 24 h. Factor levels in culture supernatants were examined by ELISA. The data are expressed as the mean ± standard errors of the means (SEM). Med. alone: medium alone; (a) ECP; (b) MBP.

The third set of experiments was undertaken to examine whether quercetin could inhibit eosinophil degranulation induced by immunological stimuli. Eosinophils were pretreated with various doses of quercetin for one hour and then stimulated with 200.0 ng/mL SCF for 24 hours. ECP and MBP contents in culture supernatants were examined by ELISA. Quercetin lower than 2.5 *μ*M scarcely affected eosinophil degranulation: the levels of both ECP (Figure 3(a)) and MBP (Figure 3(b)) in experimental culture supernatants were nearly identical (*P* > 0.05) to the control cultures. On the other hand, higher concentrations of quercetin (5.0 *μ*M and 10.0 *μ*M) significantly inhibit the degranulation induced by SCF stimulation (Figures 3(a) and 3(b)).

## 4. Discussion

Quercetin is reported to exhibit a wide variety of biological activities such as anticancer and antihypertensive effects [6]. It is also reported that quercetin inhibits mast cell activation, including inflammatory cytokine production and histamine release after immunological stimulation [8, 9, 11]. Furthermore, treatment of experimental animal models of asthma with quercetin caused not only a decrease in interleukin (IL)-5 levels and eosinophil counts in both bronchoalveolar lavage fluid and lung tissues but also bronchial hypersensitivity, which are induced by specific allergen challenge [11]. Although these reports strongly suggest that quercetin is an effective allergic inflammation suppressor, the mode of action of quercetin on allergic immune responses is not well understood.

Immunocytochemical studies of allergic diseases have shown the presence of numerous numbers of activated inflammatory cells, such as Th2 type helper T cells, macrophages, and eosinophils [15]. Of these, eosinophils are believed to be a key cell and play an essential role in the development and maintenance of allergic diseases through the secretion of lipid mediators and proteins [2, 3, 5], suggesting that eosinophils will be an important target for the treatment and the management of allergic diseases. The experiments presented here characterized firstly the effects of quercetin on allergic immune responses, especially eosinophilia and IgE hyperproduction, during normal *in vivo* immune responses. The model used is that of eosinophilia and IgE hyperproduction during *M. corti* infection. The data obtained clearly show that quercetin cannot suppress eosinophilia and IgE hyperproduction induced by *M. corti* infection, even when 20.0 mg/kg of quercetin was used for treatment. There is much evidence that eosinophilia and IgE hyperproduction caused by infection with tissue-invasive helminth species are controlled by several types of cytokines, especially IL-4 and IL-5, which are produced by Th2 type helper T cells in response to the stimulation with larval excretory/secretory antigen(s) [16–18]. The production of both IL-4 and IL-5 from Th2 type helper T cells after stimulation is also accepted to require the activation of transcription factor, such as nuclear factor-kappa B (NF-*κ*B), which controls the expression of genes encoding inflammatory cytokines [19, 20]. Although the treatment of cells with quercetin has been reported to be able to inhibit NF-*κ*B activation after inflammatory stimulation *in vitro *[21, 22], oral administration of quercetin cannot downregulate NF-*κ*B activation *in vivo *[21, 22]. Taken together, it is reasonable to speculate that the present results strongly suggest that treatment of mice with quercetin cannot suppress NF-*κ*B activation in Th2 type helper T cells induced by *M. corti* infection and that is causes the production of both IL-4 and IL-5 *in vivo*, resulting in eosinophilia and IgE hyperproduction in *M. corti*-infected, quercetin-treated mice. On the other hand, in experimental murine allergic asthma, oral administration of quercetin at a single dose of 10 mg/kg once a day for 5 days is reported to be able to suppress the increase in eosinophil numbers in both bronchoalveolar lavage fluids and peripheral blood, which are induced by relevant antigenic challenge [10]. This discrepancy may arise from the difference in models used for the experiments. Further experiments are required to clarify this point. 

Eosinophils secrete a number of harmful mediators, including ECP and MBP, which have been implicated in airway reactivity, vascular leak syndromes, destruction of epithelium, and other inflammatory changes that underlie allergic diseases [3, 5]. Eosinophils are also able to produce certain cytokines and chemokines such as IL-5, granulocyte macrophage-colony stimulating factor (GM-CSF), and RANTES which exert an autocrine effect on eosinophil survival, differentiation, and accumulation [3, 5]. SCF is a primary cytokine involved in hematopoiesis, mast cell differentiation, and mast cell activation [23]. SCF has also been shown to play a significant role in development of eosinophil-associated inflammatory responses [14, 24]. Therefore, the second part of the experiment was carried out to examine the influence of quercetin on eosinophil activation by using SCF and a cell culture technique *in vitro*. As assessed by examining the levels of RANTES and MIP-1*β*, quercetin could suppress these factor productions from eosinophils after SCF stimulation. A significant suppression of the production was firstly observed at 5.0 *μ*M. Airway mucosal eosinophilia is a prominent feature of allergic airway diseases such as asthma and rhinitis [5, 25]. Although IL-5 is accepted to be a central factor mediating eosinophil recruitment in response to allergic stimuli, chemokines, eotaxin, and RANTES are powerful chemotactic factors for eosinophils [5, 25]. MIP-1*β* is a member of the CC subfamily of chemokines, which induce the migration and recruitment of monocytes and T cells to the sites of inflammation [26]. MIP-1*β* has also been reported to enhance eosinophil effector functions by inducing the production of superoxide, which is the most important final effector molecule in inflammatory diseases [27]. In addition to the ability of eosinophils to produce these chemotactic factors, eosinophils undergo degranulation and release cytotoxic granule contents, such as ECP and MBP, which are responsible for tissue remodeling and symptoms, in response to allergic stimuli [5, 9]. Taken together, the present results showing the suppressive activity of quercetin at more than 5.0 *μ*M to produce chemokines and degranulation of eosinophils provide possible mechanisms that could explain the favorable effects of quercetin on allergic diseases. 

Although the present results clearly indicate the suppressive mode of action of quercetin on eosinophil-mediated allergic immune responses, the precise mechanisms by which quercetin could suppress eosinophil activation by SCF stimulation are not fully understood. SCF exerts its biological effect through a specific interaction with the cell surface receptor c-kit, which is a member of the receptor tyrosine kinase family [28]. SCF binding to c-kit causes the receptor to homodimerize and autophosphorylate at tyrosine residues [28]. The stimulation of c-kit leads to the activation of multiple signaling cascade (e.g., extracellular signal-regulated kinase and c-Jun N-terminal kinase, among others), which is responsible for the activation of inflammatory cells such as mast cells and eosinophils, through Ca2^+^-dependent mechanisms [28]. It is reported that quercetin could inhibit the increase in intracellular Ca2^+^ levels induced by compound 48/80 in human mast cell line *in vitro* [29], suggesting that quercetin inhibits changes in Ca2^+^ concentration in cytosol induced by SCF stimulation and results in the inhibition of release of inflammatory mediators examined. Quercetin is reported to inhibit the activation of tyrosine kinases [30–32], which are responsible for the production of several types of cytokines, including IL-4 and IL-5 [19, 20]. From these reports, there is another possibility that quercetin inhibits the activation of tyrosine kinases in eosinophils after SCF stimulation and results in inhibition of mediator release from eosinophils *in vitro.*

Dietary flavonoid glycosides are hydrolyzed in the intestine, absorbed as aglycones, and metabolized to methylated, glucurono-sulfated derivatives [33]. It is also observed that, after oral administration of 64 mg quercetin into human, hydrolyzed plasma levels of quercetin gradually increased and peaked at 650 nM and that the elimination half-life of quercetin was 17 to 24 h [34]. Commercially available preparations of quercetin recommend dosages of 400–1200 mg daily as a dietary supplement [35]. Assuming first order kinetics, a 1200 mg dose of quercetin could lead to plasma concentration up to 12 *μ*M [34], which is a much higher level of quercetin that showed suppressive effects of eosinophil activation *in vitro*. From these reports, it is strongly suggested that the findings of the present *in vitro* study may reflect the biological function of quercetin *in vivo*.

## 5. Conclusion

The results from the present study clearly demonstrate that quercetin exerts suppressive effects on eosinophil activation, but not eosinophil growth and IgE hyperproduction. These results indicate that quercetin will be a useful supplement for the management of eosinophil-mediated diseases, such as allergic rhinitis and asthma.
